# Editorial: Neurolaw: The Call for Adjusting Theory Based on Scientific Results

**DOI:** 10.3389/fpsyg.2020.582302

**Published:** 2020-10-09

**Authors:** José M. Muñoz, Eric García-López, Elena Rusconi

**Affiliations:** ^1^Department of Psychology, Universidad Europea de Valencia, Valencia, Spain; ^2^Instituto Nacional de Ciencias Penales, Mexico City, Mexico; ^3^Department of Psychology and Cognitive Science, University of Trento, Trento, Italy

**Keywords:** neurolaw, free will, criminal responsibility, psychopathy, neuroprediction, adolescent brain, addiction, decision neuroscience

The Research Topic (RT) presented here is about the complex relationship between Law and Neuroscience. We hope that it strengthens the dialogue between both disciplines across the globe, not only for a better understanding of human behavior in the legal and forensic context, but also for a better comprehension about the meaning of Justice with a view from Neuroscience. From this collection, very different positions emerge on the possible use of neuroscientific evidence to inform the law and criminal justice interventions (e.g., to safeguard personal freedom and dignity vs. to exert social control). This RT has been written by researchers from leading universities around the world and all of the papers included are based on scientific results and the most relevant and up-to-date information in each topic.

In Part I of this RT (*Neuroscience and the law: Can we fit them together?*), Pernu and Elzein advocate separating different neural-based perspectives according to their degree of viability to inform moral and legal debates on human decisions and actions, and argue that a view of neurolaw based on mixing the various perspectives is a reason for neuroscience not having strongly permeated the law as yet. Bigenwald and Chambon warn us about the possibility of a revolution in Criminal Responsibility. “Not yet” they say, by explaining what is called “the limits of Neuroscience” in their article. These limits are not only technical but legal: “[.] neuroscience can only impact legal excuses and not legal justifications.” They conclude that: “While neurolaw often evokes the neuroscientification of law, it could more properly refer to the juridification of neuroscience, i.e., legal thinking that would integrate and apply scientific discoveries to criminal justice.” Anderson and Kiehl, for their part, hold that neuroscience favors a change in normative attitudes—moving away from retributivist approaches—that, far from radically modifying the process of the legal assignment of guilt, allows it to be improved by adopting “more pragmatic strategies for combating the most conspicuous patterns promoting mass incarceration and recidivism.”

In Part II (*Neurolaw and psychopathy*), van Dongen writes about a “crucial human ability” (empathy) and focuses on the “social brain of psychopaths.” She argues that we must work on the “elucidation of the neural underpinnings of empathy” and then that we should think about “neurophysiological informed personalized treatment interventions that ultimately reduce violent transgressions in individuals with psychopathic traits.” Also, she brings an overview about psychopathy and a bio-cognitive perspective for such disorders. This second part continues with a commentary on an article by Baccarini and Malatesti (2017) in which they advocate a non-consensual application of moral bioenhancement—gene editing, neurosurgery, psychotropic treatment, etc.—to psychopaths. Their position is based on maintaining that a psychopath would allow that moral bioenhancement be applied to other psychopaths and therefore she must be treated the same way. In the commentary, Sirgiovanni and Garasic give reasons against this argument, believing that non-consensual treatment is unjustified in this case, and ultimately holding that such an invasive treatment as moral bioenhancement must be consented by the psychopath. Guillen Gonzalez et al. address the impact of biological evidence on sentencing decisions for psychopathic offenders. In a sample of German law students, evidence of brain injury but not of genetic predisposition lowered legal responsibility judgments compared to when no biological evidence was provided by the defense. No effects were found on the length of sentencing, similar to a previous study on German judges (Fuss et al., 2015) and unlike a previous study on U.S. judges (Aspinwall et al., 2012) where genetic predisposition caused the assigned prison sentence to be lowered. The authors argue that differences in criminal justice systems may explain the differential effects of biological evidence.

Tortora et al. begin Part III (*Recent advances in risk assessment*) by analyzing current evidence about how brain-reading technology—a product of the convergence between neuroimaging and AI—could be applied to forensic psychiatry and criminal justice as a tool for risk assessment and neuroprediction of violence and future recidivism. They conclude that further research must be done in this regard, and also that we would do well to anticipate debates about benefits and damages of these eventual applications of brain-reading. Haarsma et al. bring us the results of testing probationers in Houston, TX from 2017 to 2019 with a mobile neurocognitive software to predict reoffense. This NeuroCognitive Risk Assessment (NCRA) “opens the possibility of identifying different levels of recidivism risk, by crime type, for any age, or gender, and seeks to steer individuals appropriately toward rehabilitative programs.”

In Part IV (*Neuroscience and adolescent legal responsibility: The Latin American case*), Mercurio et al. address the importance of Neurobiology for the age of criminal responsibility. They argue that there is no scientific evidence to reduce the age of criminal punishment and they are “disposed not to recommend lowering the age of criminal responsibility, but rather increasing it.” This article reminds us that “Latin America does not benefit enough from the advances of the neuroscience in its application to legal issues” (García-López et al., 2019, p. 14), and also that we need that all these countries become part of this new perspective. Llamas and Marinaro draw attention to the existence of a wide range of legislative methods across Latin America concerning juvenile justice. They highlight how some of those methods may be at odds with international law and do not take into account a growing body of neuroscientific evidence showing important differences between adolescent and adult brain functioning. They advocate a revision of penological justifications in those judicial systems that still allow the application of similar punishments to juvenile offenders and adult offenders for the same crime.

Part V (*Special topics*) includes contributions on two issues that are not regularly addressed in neurolaw mainstream debates but which are relevant to make this discipline more comprehensive: moral responsibility in cases of addiction, and decision neuroscience in relation to customers' choices. Rise and Halkjelsvik present a triple study on how the different ways in which people conceive addiction cause different moral judgments when it comes to attributing responsibility to an agent. Their study showed that this attribution was “lower when addiction was connected to diseases and disorders, such as dysfunctional processes in the brain, and greater when addiction was associated with agency and addictive behaviors.” Bault and Rusconi draw attention to neuroscientifically-informed techniques that are currently used in marketing to manipulate consumer behavior and how certain groups may be particularly vulnerable to such manipulations. The growing efficacy of these techniques calls for regulatory interventions that are not limited to specific products (e.g., sweets and cigarettes) and age groups (e.g., children) but take into account our understanding of the brain circuitry for decision and of vulnerabilities to external influences, in order to preserve freedom of choice.

This RT is composed by an up-to-date collection that is both comprehensive in terms of the topics covered and balanced with regard to the inclusion of empirical, analytical, and review studies. We believe these characteristics make it a valuable tool equally useful to those interested in an introduction on neurolaw and to those looking to keep up to date with the latest and most innovative research in this fascinating and emerging discipline.

## Author Contributions

All authors contributed to the article and approved the submitted version.

## Conflict of Interest

The authors declare that the research was conducted in the absence of any commercial or financial relationships that could be construed as a potential conflict of interest.

## References

[B1] Aspinwall L. G. Brown T. R. Tabery J. (2012). The double-edged sword: does biomechanism increase or decrease judges' sentencing of psychopaths? Science 337, 846–849. 10.1126/science.1219569 22904010

[B2] Baccarini E. Malatesti L. (2017). The moral bioenhancement of psychopaths. J. Med. Ethics 43, 697–701. 10.1136/medethics-2016-103537 28356492

[B3] Fuss J. Dressing H. Briken P. (2015). Neurogenetic evidence in the courtroom: a randomized control trial with German judges. J. Med. Genet. 52, 730–737. 10.1136/jmedgenet-2015-103284 26307568

[B4] García-López E. Mercurio E. Nijdam-Jones A. Morales L. A. Rosenfeld B. (2019). Neurolaw in Latin America: current status and challenges. Int. J. Forensic Ment. Health 18, 260–280. 10.1080/14999013.2018.1552634

